# Metals and Metalloids in the Urban Segment of the Lijiang River, Guilin: Spatial Distribution, Migration and Transformation Processes, and Source Apportionment

**DOI:** 10.3390/toxics14030230

**Published:** 2026-03-08

**Authors:** Xiangru Zhang, Lianchen Zhang, Na Wu, Xiaoyun Feng, Shuyang Tan, Shuang Lü

**Affiliations:** 1 Guangxi Key Laboratory of Environmental Processes and Remediation in Ecologically Fragile Regions, Guangxi Normal University, Guilin 541004, China; zhxiangru@gxnu.edu.cn; 2 University Engineering Research Center of Green Remediation and Low Carbon Development for Lijiang River Basin, Guangxi, Guilin 541004, China; 3 College of Environment and Resources, Guangxi Normal University, Guilin 541004, China

**Keywords:** metals and metalloids, Lijiang River, sediments, source apportionment

## Abstract

The Lijiang River is a typical karst landscape river and an important drinking water source for Guilin City. To evaluate its contamination of metals and metalloids, water, surface sediment and four sediment profiles were systematically collected from the Guilin urban segment in April 2023, and the distribution, mobility and potential sources of nine elements (Cr, Mn, Co, Ni, Cu, Zn, As, Cd and Pb) were analyzed. Results show that metal and metalloid concentrations in the river water are low and water quality is good, whereas sediment concentrations of Cd, Zn, As and Pb are markedly higher than the background values. Compared with other elements, Ni, Cu, As and Cd are more readily mobilized in the aqueous phase and exhibit higher bioavailability. Vertical variation coefficients of all elements in the sediment profiles are mostly below 15%, indicating a relatively stable depositional environment. Correlation analysis and positive matrix factorization identify four main sources: industrial discharge (12.5%), mixed agricultural–geogenic origin (34.3%), traffic emissions (11.9%) and geological background (41.3%). Overall, metal and metalloid contamination in the urban Lijiang River is controllable, but accumulation of Cd and other elements in sediments requires continued attention.

## 1. Introduction

Metals and metalloids with pronounced biological toxicity, such as Cadmium (Cd), Chromium (Cr), Zinc (Zn), Copper (Cu), Arsenic (As) and Lead (Pb), are difficult to degrade in the natural environment and can persistently accumulate and migrate through the atmosphere, water bodies, and food chains, eventually entering the human body and causing lasting harm to multiple organs and systems [1,2,3,4,5]. Owing to their long-lasting persistence, formidable remediation challenges, and high restoration costs, metal and metalloid contamination has become one of the most critical environmental issues threatening public health and ecological security [6].

In river systems, metals and metalloids are distributed in the water bodies and the river sediments [7,8,9,10,11]. These elements in water can migrate with the flow and are directly linked to the ecological security of the river basin [12,13]. Approximately 85% of them are stored in sediments, which serve as a sink under stable conditions [14,15]. When environmental disturbances such as changes in water flow, redox conditions, or human activities occur, these elements can be released into the overlying water, transforming the sediment from sink to source and creating a persistent ecological threat [16,17,18]. Therefore, research on metals and metalloids in rivers must focus on both water transport and the processes of sediment accumulation and release, as these jointly determine their environmental behavior and ecological risks.

In the typical karst area of southwest China, the intricate river network interlaces surface channels with subterranean conduits, allowing pollutants to spread rapidly with the flow and expand from localized point sources into regional environmental problems [19,20,21,22]. The complexity of karst geological conditions renders the adsorption, desorption, and remobilization behaviors of metallic elements in river sediments subject to multiple environmental factors [23,24,25,26,27]. Under the influence of carbonate weathering, a weakly alkaline hydrochemical environment dominated by calcium-bicarbonate (Ca-HCO_3_) is formed, providing a favorable chemical background for metal immobilization through adsorption. Within this context, Fe-Mn oxides/hydroxides serve as primary adsorptive carriers, effectively sequestering trace elements such as As, Cd, Cr, Ni, and Pb via surface complexation, coprecipitation, and ion exchange mechanisms. Concurrently, variations in redox conditions profoundly influence metal migration and transformation behaviors, not only regulating the stability of Fe-Mn oxides but also dominating the speciation transformation of redox-sensitive elements, including Mo and Cd, where reducing environments may facilitate the dissolution and re-release of metals from oxide-bound fractions into the water column. pH fluctuations also constitute a critical controlling factor, as exemplified by storm events that can decrease water pH from 7.1 to 6.7, thereby altering metal solubility and surface adsorption site availability, and inducing the desorption and remobilization of carbonate-bound and exchangeable metal fractions. Furthermore, surface soil erosion triggered by heavy rainfall introduces substantial quantities of fine-grained sediments, which exhibit enhanced specific surface areas and adsorption capacities, thereby intensifying metal enrichment and potential bioavailability. Regular environmental monitoring of rivers in this region is therefore of great practical importance.

**Figure 1:**
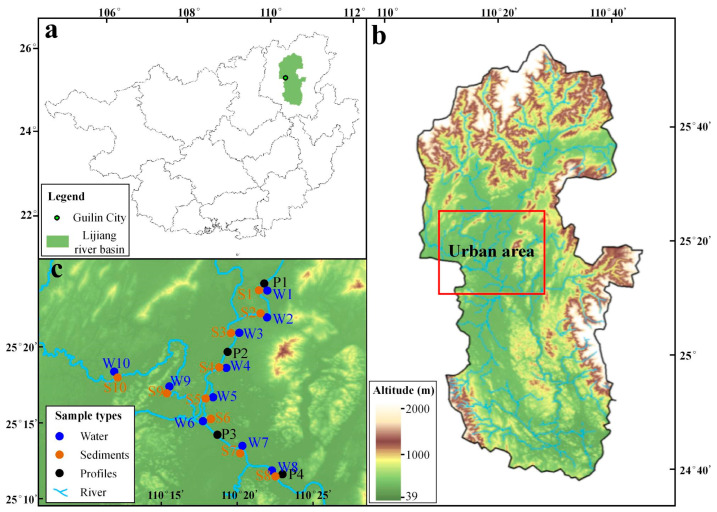
Maps showing the study area: (**a**) the location of Lijiang River Basin in Guangxi; (**b**) the topographic map of Lijiang River Basin; (**c**) the locations of sampling points.

The Lijiang River is a typical karst river in Southwest China (Figure 1). Elucidating the geochemical characteristics and behavior of metal and metalloid contaminants in its waters is essential for understanding the environmental capacity and ecological risk of such karstic systems. This study focuses on the Lijiang River, with particular emphasis on its mid-stream section flowing through the urban area of Guilin. Watershed-scale comparative studies have confirmed that this segment represents the most intensively human-disturbed area within the basin. Specifically, ecological risk assessments indicate that high-risk zones are predominantly concentrated around the main urban area of Guilin City [28], water quality monitoring results demonstrate significantly elevated pollutant concentrations in this segment [29], and studies on nitrogen species further corroborate a marked increasing trend in pollutants after the river flows through the urban district [30]. Consequently, this segment serves as an ideal study area for revealing metal and metalloid contamination characteristics, assessing ecological risks, and elucidating their migration patterns. In addition to systematically collecting water and corresponding sediment samples, this study also obtained four sediment profiles along the river, aiming to reflect the spatial distribution, migration, transformation, and pollution sources of metals and metalloids in the Lijiang River Basin from multiple dimensions. This study provides a scientific basis for water environment monitoring and management in karst river basins.

## 2. Description of the Study Area

The Lijiang River, a second-order tributary of the Pearl River system, originates from Mao’er Mountain (2141 m above sea level), the highest peak in South China, in Xing’an County, Guangxi. It flows southward through Xing’an, Lingchuan, the urban district of Guilin, and Yangshuo before converging with the Guijiang River at Pingle. With a total length of 214 km, the river drains an area of 6050 km^2^ [31]. Situated in the mid-subtropical monsoon zone, the basin experiences a mean annual temperature of approximately 18.9 °C and receives an average annual precipitation of 1949.5 mm. The topography descends progressively from an elevation of 2122 m in the northern mountainous region to 72 m in the southern valley lowlands. The northern part of the basin is underlain by clastic rocks forming medium-to-low mountain ridges, whereas the southern part is characterized by low hills and karst terrain shaped by intensive dissolution. This karst landscape, distinguished by its continuous succession of peak forests, peak clusters, and isolated peaks, along with tower-shaped landforms developed in thick, pure limestone, has been designated by the International Association of Geomorphologists as a global reference for humid tropical–subtropical karst.

As the sole municipal water source for Guilin City, the Lijiang River underpins industrial, agricultural, and domestic water supply, serving as both a critical water resource and a water quality safeguard for the region and its downstream areas [32]. The focus of this study—the Guilin urban segment—occupies the middle reach of the Lijiang River. Extending 49.3 km through a densely populated corridor, this segment functions as the primary economic and tourism hub of Guangxi, with economic activities centered on tourism and agriculture, against a backdrop of heterogeneous land use types [14,33]. Meanwhile, notable industrial activities are distributed within the study area, with the Guilin urban segment hosting numerous industrial enterprises above the designated size, encompassing industries such as electronic information, biomedicine, and high-end equipment manufacturing [14]. Owing to the inherently low resistance of karst ecosystems to external disturbances, coupled with the expansion of manufacturing and tourism in recent years, ecological pressures across the basin are intensifying [34].

## 3. Materials and Methods

### 3.1. Sample Collection

All samples were collected in April 2023 under an ambient temperature of approximately 20 °C, when the Lijiang River exhibited stable hydrological conditions, and discharge was minimally affected by rainfall.

#### 3.1.1. Sampling of Water and River Sediments

A total of 10 sites provided river water and corresponding sediment samples. The sampling sites covered the urban section of the Lijiang River’s main stem and its major tributaries. Sampling was conducted in straight river sections to avoid sewage outlets and turbulent areas, ensuring the water mass was well-mixed and representative. Water samples were mainly collected from the surface water (20 cm below the water surface) of the urban segment of the Lijiang River (Figure 1b,c). After removing foam, weeds, and other impurities, samples were stored in polyethylene plastic bottles. River sediments collected from the bottom mud (the top 10 cm of the surface layer) corresponding to the water samples were obtained using a grab sampler, and sampling sites were chosen to avoid disturbed areas to ensure the samples reflected natural deposition conditions. Then, the samples were quickly placed in clean, sealed bags, stored at low temperatures, and transported to the laboratory as soon as possible to prevent degradation or transformation of contaminants. All samples were secured in wooden boxes after field collection to prevent physical shaking during transport from affecting the elemental composition.

#### 3.1.2. Collection of Profile Samples

Four undisturbed floodplains adjacent to the main channel were selected as study sites. Prior to sampling, sufficient auxiliary tools such as polyethylene sealed bags, label paper, metal shovels, plastic rulers, and other equipment were prepared. During sampling, vegetation, gravel, and other debris on the soil surface were first removed using a metal shovel. A 50 cm deep profile was then excavated using the shovel. Starting from the surface, sediment profile samples were collected in consecutive 10 cm layers from top to bottom, with layer depths measured by a plastic ruler. For each layer, three samples were collected from left to right and mixed into a single composite sample. From each profile, five samples were collected, resulting in a total of 20 samples from four profiles. To ensure the reliability of samples, clean and uncontaminated shovels were used. After excavation, intact soil blocks that had not come into contact with the shovel surface were selected, placed into polyethylene bags, numbered, and labeled. The samples were transported back to the laboratory within 8 h after collection.

### 3.2. Chemical Analysis

The water samples, filtered through 0.45 μm membranes (Tianjin Jinteng Experiment Equipment Co., Ltd., Tianjin, China), were analyzed directly. River sediments and profile samples were air-dried at 20 °C, ground to pass through a 100-mesh sieve after removal of plant residues, and digested using a microwave-assisted system (MDS-6G) with HF-HNO_3_-HCl (Xilong Chemical Co., Ltd., Shantou, China). The digested solutions were evaporated to near-dryness, diluted to 50 g with ultrapure water, and further diluted (0.1 mL to 5 mL) for analysis by ICP-MS (Agilent 8900, manufactured in Santa Clara, CA, USA, 2015) of Cr, Mn, Co, Ni, Cu, Zn, As, Cd and Pb. All reagents were analytical grade, and labware was pre-cleaned in concentrated HNO_3_. Ultrapure water blanks (every 12 samples) and certified reference materials (GSR-1 and GSR-3) were used for quality control to ensure all the results were within acceptable limits.

### 3.3. Data Processing

#### 3.3.1. Health Assessment Model

This study employed the US EPA water-related human health risk model [35] to quantify the total health risk (RT) posed to humans through the drinking water pathway. RT is the sum of the carcinogenic risk induced by the carcinogenic metals (Rc) As, Cr and Cd, and the non-carcinogenic risk (Rn) arising from the non-carcinogenic elements Pb, Cu, Co, Zn and Mn:

RT = Rc + Rn

Rc = Σ R^i^c = Σ [1– exp (–Di × Qi)]/72

Rn = Σ R^i^n = Σ (Di/RFDi) × 10^−6^/72

Di_(for adults)_ = 2.2 × Ci/64.3

Di_(for children)_ = 1.0 × Ci/22.9

Here, Ci is the mass concentration of element i (mg/L), Di denotes the average daily exposure dose per unit body weight for toxic substance i (mg/(kg·d)), Qi is the carcinogenic potency coefficient of carcinogenic element i through the drinking water pathway ((kg·d)/mg), while RFDi is the reference dose for the ingestion of non-carcinogenic element i by humans via the drinking water pathway (mg/(kg·d)) [35]. “72” represents the average life expectancy in Guangxi (years), “2.2” and “1.0” are the mean daily water ingestion rates for adults and children (L/d), and “64.3” and “22.9” are the corresponding average body weights (kg). When RT < 1.0 × 10^−6^ a^−1^, it is classified as a low risk level. The above-mentioned regional average life expectancy, water intake, body weight, and ecological risk classification in Guangxi refer to the published literature [36] in this region.

#### 3.3.2. Environmental Risk Assessment

The environmental risk assessment of metals and metalloids in sediments is conducted using the Potential Ecological Risk Index (PERI) method [37]. The calculation formula is as follows:

E^i^r = Tir × C^i^r/C^i^n

RI = Σ E^i^r = Σ (T^i^r × C^i^r/C^i^n)

In the formula, RI represents the comprehensive potential ecological risk index of elements; E^i^r denotes the potential ecological risk index of element i at sampling point r; T^i^r is the corresponding toxicity coefficient of element i; C^i^r refers to the measured concentration of element i; and C^i^n represents the reference value for element i. In this paper, the selected reference values are from reference [14]. E^i^r below 40 means low ecological risk, and it climbs through moderate, considerable and high until it reaches very high at 320 or above, while RI below 150 signals low risk and it ascends through the same verbal thresholds until it hits very high at 1200 or more [37].

#### 3.3.3. Coefficient of Variation

The coefficient of variation (CV) is a normalized statistical measure of data dispersion, used to gauge stability and fluctuation. It is calculated as

CV = σ/μ × 100%

where σ is the standard deviation and μ is the mean. CV indicates the extent to which element concentrations have been influenced by anthropogenic activities; in this paper, the variability is classified as weak when CV < 15%, moderate when 15% ≤ CV ≤ 35%, and strong when CV > 35%. This methodological approach, including the classification thresholds, is consistent with previous regional studies [38], allowing for comparability of results.

#### 3.3.4. Distribution Coefficient

The partitioning of each element between water and sediment is described by the distribution coefficient:

Kd = C_sediment_/C_river_

where C_river_ and C_sediment_ are the metal concentrations in river water and sediment, respectively. A lower Kd indicates that the element tends to remain in the aqueous phase, whereas a higher Kd implies greater affinity for sorption onto sediment. In the context of a river system, this principle has been widely applied to describe the partitioning of elements between water and sediment [39].

#### 3.3.5. Positive Matrix Factorization

The Positive Matrix Factorization (PMF) model is widely used to apportion potential sources of metals and metalloids in various environmental media and to quantify their interrelationships [40]. In this study, EPA PMF 5.0 is employed. The workflow is as follows: first, the analytical uncertainty of each element concentration is estimated; the concentration matrix and its corresponding uncertainty file are then loaded into the model, and the number of factors is varied to represent distinct pollution sources. Optimization showed that with 13 iterations and a seed value of 22, the robust Q value reached its minimum, residuals are almost all within ±3, and individual R^2^ for every element ranged from 0.88 to 0.99, confirming high model fit and reliable results.

## 4. Results

### 4.1. The Metal and Metalloid Concentration of the Water

Concentrations of nine elements (Cr, Mn, Co, Ni, Cu, Zn, As, Cd, Pb) in the Lijiang River water are summarized in Table 1. The observed ranges are 0.098–0.469 for Cr, 0.158–1.321 for Mn, 0.038–0.111 for Co, 0.301–0.684 for Ni, 0.257–0.550 for Cu, 0–1.995 for Zn, 0.590–1.292 for As, 0.012–0.067 for Cd, and 0–0.023 for Pb. Zn exhibits the widest concentration span, whereas Ni shows the most restricted distribution. Coefficients of variation (CV) exceed 1 for Pb and Zn, indicating strong spatial variability; CVs for Cr, Mn, Co, Ni, Cu, As, and Cd range from 0.2 to 0.8, reflecting moderate variability.

### 4.2. Metal and Metalloid Concentration of the River Sediments

Average concentrations of Cr, Mn, Co, Ni, Cu, Zn, As, Cd and Pb in the corresponding river sediments are 60.7, 569.4, 13.2, 26.8, 34.8, 111.2, 19.7, 0.88 and 30.0 mg/kg, respectively (Table 2). The quality assessment of the aforementioned river sediments is based on sediment quality guidelines (SQGs) [42,43], which define critical concentration thresholds associated with specific effects on biota. Specifically, the threshold effect concentration (TEC) represents the minimal effect boundary, where adverse ecological effects are expected to occur only rarely when sediment concentrations fall below this value; the probable effect level (PEL) represents the significant effect boundary, above which adverse effects are anticipated to occur frequently, posing a severe threat to organisms; concentrations between TEC and PEL fall within the possible effect range, indicating that adverse effects may occasionally manifest. In this study, except for As at individual sites, all other elements remained below PEL, suggesting that the overall sediment quality in the study area is controllable and has not yet reached pollution levels that would cause severe adverse effects on biota. However, all elements exceeded TEC at certain locations, particularly Cr and As, which were above TEC at all sampling sites, indicating that these elements may pose ecological risks under specific regional or conditional circumstances, warranting continuous attention and enhanced monitoring and prevention measures. Coefficients of variation for the nine elements range from 0.15 to 0.5, indicating moderate spatial variability.

### 4.3. Variations in Metal and Metalloid Concentration of the Sediment Profiles

The results of the metal and metalloid concentrations in profiles are shown in Table 3. The concentrations of all elements in the profiles are comparable to those in river sediments.

**Figure 2:**
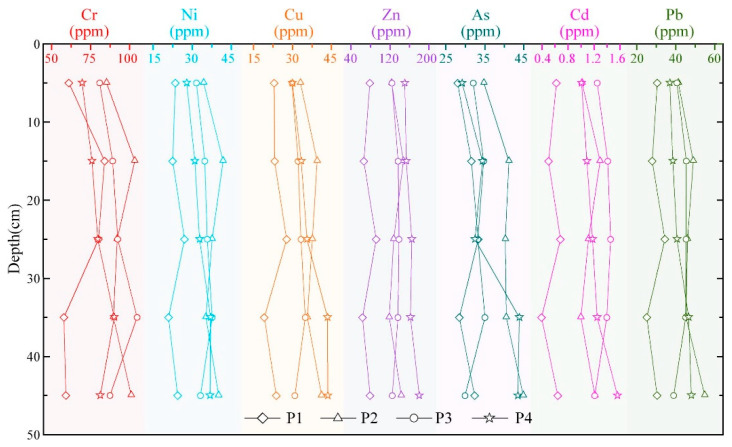
Variation in metal and metalloid concentration along the sediment profiles.

Spatially, Cr and Ni exhibit coincident accumulation peaks, both reaching maxima at 10–20 cm in profiles 1 and 2 and at 30–40 cm in profiles 3 and 4. In contrast, Cu and Zn maxima are more scattered, occurring at different depths in each profile. Cd and Pb display highly concordant patterns, with a pronounced mid-profile enrichment. Vertically, all metals follow a generally similar trend: from bottom to top, concentrations decline from 50–40 cm, rise at 40–30 cm, and then decrease steadily toward the surface; the overall maxima are recorded in the 30–20 cm interval. Throughout the cores, elemental variations remain modest, indicating a relatively stable history of deposition in the study area (Figure 2).

## 5. Discussion

### 5.1. Composition and Distribution Characteristics of Metals and Metalloids in the Water

**Figure 3:**
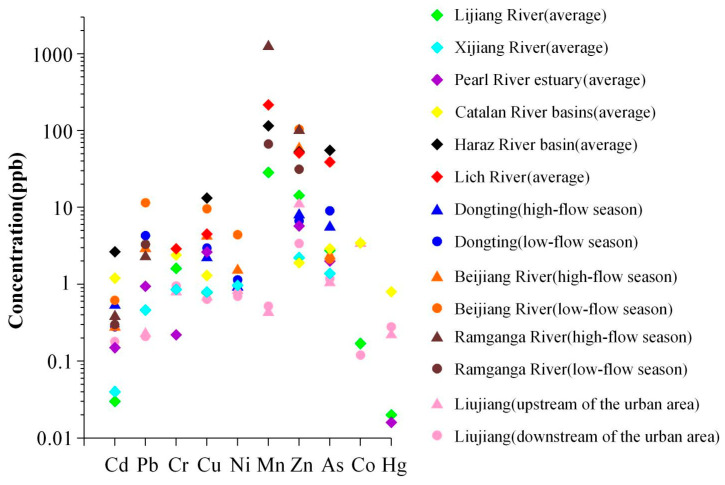
Differences in metal and metalloid concentrations of different rivers at various intervals. The average data of Lijiang River, Xijiang River, Pearl River estuary, Catalan River basin, Haraz River basin and Lich River are sourced from References [36,46,47,48,49,50] respectively; the seasonal data of Dongting, Beijiang River and Ramganga River are from References [11,51,52], respectively; and the data of the Liujiang is from Reference [53].

Metals and metalloids in surface water occur predominantly in dissolved and suspended forms, with dissolved fractions generally exceeding suspended concentrations [44,45]. Owing to differences in catchment geochemistry, elemental concentrations vary markedly among rivers (Figure 3), and pronounced spatial–temporal heterogeneity is commonly observed along the same river between upper, middle and lower reaches and between wet and dry seasons [11,16,19,33,36,46,47,48,49,50,51,52,53]. The concentrations of elements such as Zn, Co, and Mn fluctuated considerably across different river sections and seasons. This is likely attributable to variations in the hydrogeological background and the intensity of human activities along different segments of the river. Their high spatial and temporal variability indicates that these elements are susceptible to external environmental influences. The overall metal and metalloid concentration in the water of the Lijiang River is lower than that in other typical regional rivers in China, which may be related to the stricter environmental control measures implemented in Guilin as an international tourist city. Spatially, however, the urban segment that traverses the city core exhibits significantly higher metal levels than both upstream and downstream sections, indicating a localized hotspot of anthropogenic influence, primarily driven by industrial and agricultural effluents and urban diffuse pollution [19,33,36].

**Figure 4:**
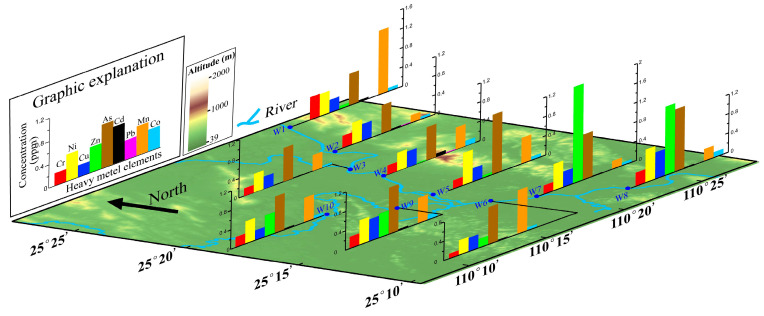
Metal and metalloid distribution of water bodies in urban segment of Lijiang River.

Figure 4 illustrates the spatial heterogeneity of the metals and metalloids at ten water sampling sites. Ni, Cu and Co are distributed relatively uniformly; by contrast, Pb, Zn, As, Cr, Cd and Mn differ markedly among sites. Pb was detected only at W2 and W4, and Zn showed anomalously high values at W7 and W8. Given that their coefficients of variation exceed 1, Pb and Zn are likely derived from point source pollution. The anomalously high Zn values at W7 and W8 may indicate anthropogenic pollution. In addition, concentrations of Cr, Cd and Mn at site W1, which is close to villages and farmland, are all high, revealing a strong influence of human activities. Compared with 2013, the concentrations of all elements in the urban segment of the Lijiang River have remained basically stable, with no significant overall change [19].

Compared with the Environmental Quality Standards for Surface Water (GB 3838-2002) [41], all indicators of these elements in the urban segment of the Lijiang River are better than the Class I surface water limits, reflecting excellent water quality conditions. The US-EPA health risk model further indicates that the combined health risk faced by both adults and children exposed through the drinking water pathway remains low (Table 4).

Nevertheless, all toxic elements pose a distinctly greater potential health threat to children than to adults. Among carcinogenic elements, the mean individual health risk decreases in the order As > Cr > Cd; whereas for non-carcinogenic metals, the sequence is Cu > Co > Pb > Zn > Mn. Notably, the maximum average individual annual health risks calculated by this model for As and Cr are both 1.17 × 10^−5^/a which is below the maximum acceptable level recommended by the International Commission on Radiological Protection (ICRP) (5.0 × 10^−5^/a) but above the maximum acceptable risk levels recommended by the Swedish Environmental Protection Agency, the Dutch Ministry of Housing, the Spatial Planning and the Environment, and the UK Royal Society (1.0 × 10^−6^/a), indicating a certain carcinogenic risk [36]. Therefore, although the Lijiang River still retains environmental capacity for metal and metalloid contamination, long-term trends in As, Cr and similar contaminants must be kept under continuous monitoring and scrutiny. It should be noted that the health risk assessment in this study is based on the principles of protectiveness and prevention, adopting a conservative assumption that the water body under investigation is treated as a potential source for direct daily drinking. However, in reality, drinking water for residents typically undergoes treatment at water treatment plants, and the actual exposure level is also closely related to residents’ water usage habits. If the water body is not actually used as a source of drinking water, the actual health risk level will be significantly lower than the estimates provided in this study.

### 5.2. Migration and Transformation Patterns of Metals and Metalloids in River Systems

Despite the low concentrations of metals and metalloids in the water bodies, a significant accumulation of these metals is observed in the sediments of the river system (Table 1 and Table 2). The concentrations of all elements in the sediments are approximately 10^4^ times higher than their corresponding concentrations in the water, indicating that the metals and metalloids in the river system are predominantly stored in the river sediments. Based on estimates of the total water volume and surface sediment mass in the Lijiang River, approximately 85% of the metals and metalloids are retained in the surface sediments [14,15]. Analysis of the distribution coefficients (Kd) of elements between water and river sediments reveals that As, Cd, Ni and Cu exhibit lower Kd values compared to Co, Cr, Zn, Mn and Pb (Table 5). This suggests that As, Cd, Ni and Cu are more likely to remain in the aqueous environment and migrate with the water flow, thereby posing higher bioavailability and potential ecological risks.

Sediments are generally viewed as a quasi-inert reservoir of metals and metalloids whose reactivity is far lower than that of the dissolved phase; consequently, they are assumed to pose only minor immediate stress to the catchment ecosystem. However, pronounced shifts in redox potential, pH, or sediment grain size can trigger re-mobilisation of elements from the solid phase, creating a latent threat to downstream ecological security [19]. Research on the Narmada River (India) indicates that when the river’s pH exceeds 8, the alkaline environment can promote the dissolution of elements such as Mn, Cd, and Cr or alter the surface properties of colloids, thereby enhancing the release potential of metals and metalloids [8]. Fluctuations in oxidation–reduction potential influence the migration behavior of elements by changing their valence states and forms of occurrence. This is particularly significant for metals with multiple oxidation states, such as Mn and Cr. For example, in the sediments of the Musa Estuary in the northwestern Persian Gulf, Mn mainly exists in a reducible state, exhibiting a high risk of migration and release under fluctuating redox potential [54]. Sediment particle size regulates elemental enrichment through the particle size effects of specific surface area and cation exchange capacity [55]. Fine particles (silt-clay) preferentially adsorb metals and metalloids due to their larger specific surface area and stronger cation exchange capacity [56]. In high-energy river environments like the Estrela River in Brazil, the loss of fine particles caused by hydrodynamic conditions weakens this effect, resulting in lower metal and metalloid enrichment levels in systems dominated by sandy sediments [57]. Additionally, in rivers, organic matter forms stable complexes with ions through its abundant functional groups (e.g., carboxyl and hydroxyl groups) [58]. High organic matter content typically enhances the adsorption and fixation capacity for metals and metalloids, reducing their environmental risk, and low molecular weight organic matter may promote the dissolution and migration of these elements.

**Figure 5:**
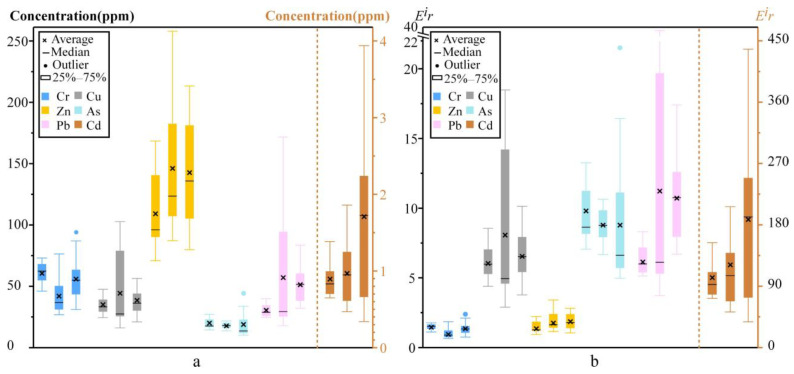
Variations in metal and metalloid concentration (**a**) and ecological risk (**b**) in the river sediments of the Lijiang River. For each element (from left to right), three data groups were obtained in 2023 (this study), 2019 [14], and 2013 [19], respectively.

To evaluate the long-term behavior of individual metal and metalloid concentrations and associated ecological risks of Cr, Cu, Zn, As, Pb, and Cd in river sediments from the urban segment of the Lijiang River are compared across three sampling campaigns in different years (Figure 5).

The concentrations of all metals remain stable and consistent over the ten-year interval, with no significant directional change observed in their temporal patterns (Figure 5a). Both the single potential ecological-risk coefficient (E^i^r) for each element and the integrated potential ecological risk index (RI) show no significant differences across the three sampling campaigns, showing a low to moderate risk level. Cadmium, owing to its high toxic-response factor, contributed most to RI, yet its absolute concentration and corresponding E^i^r value also remained stable (Figure 5b). This result indicates that, despite anthropogenic disturbances within the watershed, the metal and metalloid concentration of the sediments in the study segment has not undergone significant changes over the past decade, demonstrating a relatively stable function as an inert reservoir. This suggests that a dynamic equilibrium may have been reached between external inputs and the sediment’s retention capacity. This stability is closely linked to a series of protective measures implemented following the promulgation of the Regulation on the Ecological and Environmental Protection of the Lijiang River Basin in the Guangxi Zhuang Autonomous Region (2011), including the closure of quarries and the strengthening of nature reserve construction and management. As industrial activities in the Lijiang River Basin have gradually declined and the ecological environment has continuously improved, the variation in elemental concentrations in sediments has leveled off over the past decade, with no significant fluctuations observed. This reflects that the environmental quality of the basin has entered a relatively stable state. Additionally, four 50 cm profiles reveal vertical coefficients of variation for all elements of <15% (Figure 2; Table 3), signifying homogeneous down-core distributions and the absence of discrete contamination events. Nevertheless, total metal and metalloid concentration is insufficient to assess bioavailability or environmental risk, so sequential extraction (BCR) is required to resolve metal speciation in future work.

### 5.3. Source Apportionment of Metals and Metalloids in the Urban Segment of the Lijiang River

To further elucidate the interrelationships and potential sources of metals and metalloids in the Lijiang River, correlation analysis and positive definite matrix factorization (PMF) are jointly applied to the sediment dataset.

**Figure 6:**
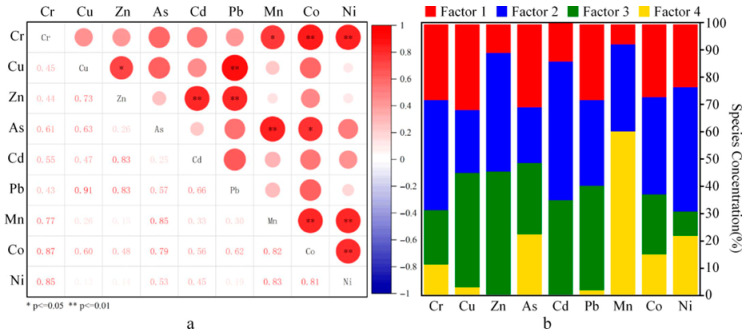
Correlation analysis of metal and metalloid elements in sediments (**a**) and source contribution signatures of the four PMF-derived factors (**b**).

The correlation analysis (Figure 6a) results indicate that Cu exhibits a strong correlation with Pb (r = 0.91), while Co, Cr, Ni, and Mn also display a high degree of coupling (r > 0.7), suggesting that these elements likely share a common origin. Additionally, significant correlations between Cd and Zn (r = 0.83), and between As and Co, Cr, Cu (with r values of 0.79, 0.61, and 0.63, respectively), further imply similar geochemical sources of these elements. Based on the PMF model, the sources of metals and metalloids can be resolved into four factors (Figure 6b), with a cumulative contribution rate of 100%. Factor 2 (34.3%) and Factor 4 (41.3%) emerge as the primary contributors, collectively accounting for 75.6% of the total contribution source. The interpretation of each factor is as follows:

Factor 1 accounts for 12.5% of the total contribution and exhibits high loadings for Cu (32.5%), As (30.4%), Pb (28.3%), and Cr (28.1%). The concentrations of these elements all exceed, to varying degrees, the corresponding sediment background values, indicating a certain level of contamination. Specifically, the enrichment of Cr and As may be directly related to industrial point-source emissions, such as those from coking plants and electroplating facilities along the river [19]. The accumulation of Cu and Pb may also originate from industrial waste gas and wastewater discharges [59]. Therefore, Factor 1 is identified as an industrial source.

Factor 2 is the second largest contributing source, showing significant contributions to Cd (50.9%), Ni (46.0%), Zn (44.0%), Cr (40.9%), and Co (35.8%). In anthropogenic activities, Cd and Zn are commonly associated with agricultural practices. For instance, Cd is linked to the use of chemical fertilizers and pesticides [60], while Zn may also originate from fungicides and insecticides [61]. The urban segment of the Lijiang River is influenced by long-term agricultural development in history, with farmland being widely distributed along its banks [33]. During cultivation, these farmlands may introduce a certain amount of elemental pollutants into the river water through surface runoff and soil leaching. Therefore, Factor 2 is likely related to agricultural activities. Additionally, in karst regions, weathering of parent rocks and soil-forming processes can lead to the enrichment of Cd in soils [62,63]. The exceptionally high Cd content in the Lijiang River sediments may thus be influenced by both anthropogenic activities and natural geological processes. In contrast, the contents of Ni, Co, and Cr are relatively low, falling below regional background levels, which is often indicative of natural background sources [14]. Consequently, Factor 2 can be interpreted as reflecting combined influences from agricultural activities and geological background.

Factor 3 accounts for 11.9% of the total contribution and primarily loads on Zn (45.8%), Cu (42.1%), Pb (38.4%), and Cd (34.7%). This elemental combination is commonly associated with traffic emissions, resulting from processes such as fuel combustion, tire and brake wear, and lubricant usage [19,64]. Frequent tourist cruise ship activity occurs in the urban segment of the Lijiang River, where the fuel combustion and mechanical wear from these vessels may contribute to inputs of Cd and Zn [15]. Considering the regional environmental characteristics, Factor 3 is likely indicative of traffic-related emissions.

Factor 4 demonstrates the highest contribution rate, with a particularly prominent loading on Mn (60.0%), while also showing certain contributions to As (22.2%), Ni (21.8%), and Co (14.8%). Mn is typically closely associated with geological sources, such as rock weathering and the formation of Fe-Mn nodules [62]. Ni and Co are also predominantly derived from natural background sources [14]. Therefore, Factor 4 primarily reflects a geological background source.

In summary, the high correlation clusters identified by correlation analysis, namely Cu-Pb, Cd-Zn, and Co-Cr-Ni-Mn (r > 0.7, *p* < 0.01), are predominantly contributed to by the same major factors in the PMF model. For instance, Cu and Pb are jointly influenced by Factor 1 and Factor 3, while Cd and Zn are jointly governed by Factor 2 and Factor 3, with each corresponding factor contributing over 30% to the relevant elements, indicating their common sources. Meanwhile, the moderate correlations observed between As and multiple elements in the correlation matrix are quantitatively decomposed in the PMF model as dual loadings on Factor 1 (industrial-agricultural source) and Factor 4 (geological background source), further explaining its association with various environmental processes. The above findings demonstrate that the correlation heatmap and PMF source apportionment results corroborate each other, jointly revealing that the sources of metals and metalloids in the study area are characterized by the combined influence of anthropogenic emissions and natural processes.

## 6. Conclusions

This study systematically analyzed the distribution, migration and transformation characteristics, and potential sources of nine metals and metalloids (Cr, Mn, Co, Ni, Cu, Zn, As, Cd, Pb) in water, surface sediments, and sediment profiles from the urban segment of the Lijiang River in Guilin. The results indicate that the water quality in this segment is excellent, posing a low potential risk to human health. However, elements such as Cd, Zn, As, and Pb show significant enrichment in sediments, with concentrations notably higher than background values. In terms of migration behavior, Ni, Cu, As and Cd tend to migrate in the aqueous phase, exhibiting higher bioavailability and environmental activity. Sediment profile data reveal a relatively uniform vertical distribution of metals and metalloids, with no significant fluctuation in ecological risks over the past decade, suggesting an overall stable sedimentary environment in the area. Source identification further indicates that metals and metalloids in the study area are primarily influenced by natural geological background, while contributions from anthropogenic factors such as industrial emissions, agricultural activities, and traffic sources cannot be overlooked. In summary, metal and metalloid contamination in the urban segment of the Lijiang River is currently under overall control. Nevertheless, continuous attention should be paid to the accumulation of elements such as Cd in sediments, and targeted management and control of anthropogenic pollution sources should be strengthened to ensure the long-term safety of the riverine ecosystem.

## Figures and Tables

**Table 1:** Statistical analysis of metal and metalloid concentrations in water.

	Max	Min	Mean	SD	CV	Class I of the Environmental Quality Standards for Surface Water [41]
Cr	0.47	0.098	0.23	0.11	49.30	10.00
Mn	1.32	0.16	0.51	0.41	78.80	—
Co	0.11	0.038	0.07	0.027	37.90	—
Ni	0.68	0.30	0.49	0.14	28.60	—
Cu	0.55	0.26	0.33	0.095	29.10	10.00
Zn	2.00	—	0.47	0.78	168.50	50.00
As	1.30	0.59	0.86	0.27	31.70	50.00
Cd	0.067	0.012	0.028	0.018	64.50	1.00
Pb	0.023	—	0.005	0.009	189.10	10.00

The standard deviation (SD) is unitless, the coefficient of variation (CV) is %, and the units for the other indicators are μg/L.

**Table 2:** Metal and metalloid concentration in the river sediments.

	Max	Min	Mean	SD	CV	TEC [42]	PEL [43]
Cr	73.03	46.07	60.71	9.49	15.70	43.4	90
Mn	1008.26	266.50	569.41	259.91	47.40	460	-
Co	14.62	9.20	13.15	2.04	17.40	50	-
Ni	35.69	18.70	26.78	6.32	23.80	22.7	-
Cu	47.63	24.44	34.81	8.13	23.20	31.6	197
Zn	168.44	70.93	111.17	33.92	29.90	121	315
As	27.19	14.48	19.70	4.19	21.60	9.8	17
Cd	1.39	0.65	0.88	0.24	26.10	0.99	3.5
Pb	39.93	24.64	30.02	5.10	16.90	35.8	91.3

The standard deviation (SD) is unitless, the coefficient of variation (CV) is %, and the units for the other indicators are mg/kg. PEL—probable effect level; TEC—threshold effect concentration.

**Table 3:** Characteristics of metal and metalloid concentration in profile.

Sampling Site		Cr	Ni	Cu	Zn	Cd	Pb
P1	Max	83.80	27.08	27.76	91.28	0.68	34.42
	Min	57.79	20.91	19.15	63.44	0.39	25.18
	Mean	68.29	23.68	23.33	75.65	0.57	30.10
	CV	16.4	6.70	9.47	10.76	15.06	6.71
P2	Max	103.18	41.90	41.18	147.00	1.29	54.830
	Min	85.14	34.43	33.07	118.33	1.00	41.35
	Mean	94.13	37.90	37.42	130.39	1.13	47.28
	CV	7.28	5.53	6.25	6.74	9.71	8.75
P3	Max	104.67	37.69	34.94	138.27	1.46	45.39
	Min	80.73	31.67	29.83	123.91	1.21	38.90
	Mean	90.80	34.43	32.22	131.84	1.35	43.05
	CV	7.04	4.70	4.70	2.90	5.50	5.53
P4	Max	90.05	37.05	43.57	179.70	1.56	47.96
	Min	69.47	27.90	29.96	150.17	1.01	36.95
	Mean	79.17	33.13	37.18	161.85	1.22	42.12
	CV	7.73	8.75	15.04	4.01	17.06	10.51

The coefficient of variation (CV) is %, and the units for the other indicators are mg/kg.

**Table 4:** The average annual health risk of metal and metalloid contaminants to adults and children.

		Carcinogenic Elements			Non-Carcinogenic Elements				
		As (10^−6^)	Cr (10^−6^)	Cd (10^−7^)	Cu (10^−11^)	Zn (10^−12^)	Pb (10^−12^)	Mn (10^−13^)	Co (10^−12^)
Adults	Range	4.20~9.21	1.91~9.13	0.35~1.94	2.44~5.23	0~3.16	0~7.81	0.54~4.48	4.51~13.2
	Average	6.11	4.44	0.80	3.12	0.74	1.65	1.74	8.32
	Rc	3.15 × 10^−6^			-				
	Rn	-			8.41 × 10^−12^				
	Risk level	Low risk							
Children	Range	5.37~11.7	2.44~11.7	0.44~2.48	3.12~6.67	0~4.03	0~9.96	0.68~5.72	5.76~16.8
	Average	7.08	5.66	1.02	3.98	0.94	2.11	2.22	10.6
	Rc	4.52 × 10^−6^			-				
	Rn	-			8.50 × 10^−11^				
	Risk level	Low risk							

The unit of the data in the table is year (a).

**Table 5:** The distribution coefficients of the metal and metalloid elements (Log (Kd)).

	Cr	Ni	Cu	Zn	As	Cd	Pb	Mn	Co
Range	5.01~5.82	4.47~4.96	4.73~5.28	4.85~6.68	4.11~4.61	4.04~5.06	6.09~6.19	5.30~6.68	4.92~5.51
Mean	5.45	4.74	5.03	5.70	4.39	4.54	6.14	6.09	5.25

## Data Availability

The original contributions presented in this study are included in the article. Further inquiries can be directed to the corresponding author.
